# Barriers and enabling factors for utilizing physical rehabilitation services by Afghan immigrants and refugees with disabilities in Iran: a qualitative study

**DOI:** 10.1186/s12889-024-18374-4

**Published:** 2024-03-25

**Authors:** Elaheh Amini, Manal Etemadi, Saeed Shahabi, Cornelia Anne Barth, Farzaneh Honarmandi, Marzieh Karami Rad, Kamran Bagheri Lankarani

**Affiliations:** 1 The International Committee of the Red Cross, Tehran Delegation, Tehran, Iran; 2 The National Institute for Health and Care Research Applied Research Collaboration West (NIHR ARC West) at University Hospitals Bristol and Weston NHS Foundation Trust, Bristol, UK; 3 Population Health Sciences, Bristol Medical School, University of Bristol, Bristol, UK; 4 Health Policy Research Center, Institute of Health, Shiraz University of Medical Sciences, Shiraz, Iran; 5 Centre for Primary Care and Public Health (Unisanté), University of Lausanne, Lausanne, Switzerland; 6 Student Research Committee, Shiraz University of Medical Sciences, Shiraz, Iran

**Keywords:** Rehabilitation, Disability, Immigrants, Refugees, Qualitative study, Iran

## Abstract

**Introduction:**

Individuals with a migrant background often underutilize physical rehabilitation services (PRS) compared to the host population. This disparity is attributed to various barriers, including limited access to information, language barriers, illiteracy, and cultural factors. To improve PRS utilization by Afghan immigrants and refugees in Iran, it is crucial to identify these barriers and enabling factors. In response, this study explored the barriers and enabling factors for utilizing PRS among Afghan immigrants and refugees with disabilities in Iran.

**Methods:**

This qualitative study was conducted in Iran between January and March 2023. Participants were selected through convenient and snowball sampling. Individual, semi-structured interviews were carried out both in face-to-face and online formats. Data analysis occurred concurrently with data collection, using the directed content analysis approach.

**Results:**

Findings from our research indicate that common barriers to PRS utilization among Afghan immigrants and refugees include insufficient insurance coverage, high service costs, expensive transportation and accommodation, limited knowledge about Iran’s health system, inadequate awareness of available supports, restricted access to PRS in remote areas, impatience among PRS providers, fear of arrest and deportation, a lack of trust in modern treatments, stringent immigration rules, high inflation rates limiting the ability to pay for PRS, and limited social support. On the other hand, several enabling factors were identified, such as strengthening insurance coverage, utilizing the capacities of charities and NGOs, providing information about available services, promoting respectful behavior by healthcare providers towards patients, facilitating cultural integration, and increasing immigrants’ awareness of available services and eligibility criteria.

**Conclusion:**

The barriers and enabling factors uncovered in this study offer valuable insights into the complexities surrounding PRS utilization by Afghan immigrants and refugees with disabilities in Iran. Understanding and addressing these factors is essential for developing targeted interventions and policies that can improve access and utilization, ultimately leading to enhanced health outcomes for this vulnerable population.

## Introduction

The movement of people across country borders affects the host country in varies ways, influencing labor markets, productivity, innovation, demographic structure, fiscal balance, and criminality [1]. Population mobility serves as a cornerstone for meeting the labor and economic demands for human capital. It also helps mitigate the social, demographic, and economic impacts of aging populations in many economically advanced nations where increased migration is required to sustain labor markets and population growth [2, 3]. Notably, a percentage of refugees can bring valuable skills to the labor market. The IMF estimated that 21% of Syrian asylum seekers who arrived in Germany between 2013 and 2014 reported having a tertiary education [4]. Similarly, immigrants contribute significantly to healthcare occupation, with 15.6% in healthcare practitioners and technical roles and 22.4% in healthcare support positions in the US [5].

Conflict and displacement can increase the risk of disability either directly, caused by war-related trauma and injuries or indirectly through the breakdown of health systems [6]. These risks may be especially common in situations of displacement where there can be varying levels of access to health care in host countries, which causes and/or further exacerbates disability [7]. The World Health Organization (WHO) estimates that 2.4 billion people have health conditions that would benefit from rehabilitation services [8]. UNHCR estimates that there will be 70.8 million people forcibly displaced worldwide by the end of 2018 [9]. Accordingly, almost 7.65 million people with disabilities would face forced displacement [10]. Refugees and asylum seekers with disabilities are confronted with multiple and intersecting forms of discrimination and have worse health outcomes [11].

Studies estimate that one in six refugees has a physical health problem severely affecting their lives, and two-thirds experience mental health problems [12]. There is a high prevalence of non-communicable diseases (NCDs) in the refugee population, resulting in high levels of vulnerability and long-term health complications [13, 14]. Many refugees also have pre-existing disabilities and chronic health conditions. These conditions underpin the important role of physical rehabilitation services (PRS) [15]. Immigrants and refugees with disabilities do not have equitable access to resettlement opportunities, and some countries put restrictions on the immigration of individuals with intellectual disabilities, partially due to the cost of needed educational and health services. These refugees face more marginalization and cumulative disadvantage [16]. Consequently, people from refugee backgrounds with disabilities are likely to simultaneously encounter the dual disadvantages associated with being both a refugee and a person with a disability [17].

People with a migrant background are less likely to access PRS compared to those without, possibly due to barriers such as lack of information, language problems, illiteracy, and cultural aspects [18–21]. Additionally, health insurance coverage has been found to be a potential predictor of immigrant health service utilization, and low income appears to be a barrier to the use of secondary care [22]. Furthermore, transport, stigma and discrimination, and financial and social isolation are among the common barriers to accessing services among immigrants’ families with disabilities [23, 24]. Importantly, people with migrant backgrounds and foreign nationals faced more barriers to accessing care and poorer rehabilitation outcomes due to a lack of culture- or gender-sensitive treatment concepts among health care providers [25].

In Bangladesh, the lack of PRS and inclusive programs, along with a difficult and inaccessible landscape and the stigmatization of people with physical, mental, and psychological disabilities, make it hard for people with disabilities (PwD) to use their basic human rights and fully participate in their community [26]. Refugees from Syria with disabilities living in Turkey face particular challenges, including stigma, in securing income-generating opportunities [27]. Also, Norredam et al. (2010) concluded that problems of access for migrants may be related to formal and informal barriers. User fees, organizational barriers, and a lack of referral between services are just a few examples of the elements that make up formal barriers in the health system. Other factors include legal restrictions on access for specific groups. On the other hand, language, communication, socio-cultural factors, and ‘newness’ may lead to informal barriers affecting migrants’ utilization patterns [28]. These studies have enhanced the understanding of the challenges faced by PwD with a migrant background in accessing essential services, with a particular focus on PRS.

In order to facilitate the utilization of PRS by Afghan immigrants and refugees in Iran, the identification of barriers and enabling factors is crucial. This information can inform national and international organizations and authorities on effective strategies to ensure immigrants’ access. Notably, it is worth acknowledging that, to the best of our knowledge, there has been a notable gap in existing research regarding the utilization of PRS by Afghan immigrants and refugees with disabilities in Iran. In light of this knowledge gap, our research team initiated a qualitative study aimed at identifying the barriers and enabling factors for this vulnerable group to utilize PRS.

## Methods

This qualitative study was carried out in Iran from January to March 2023, with the participation of immigrants and refugees in need of rehabilitation and also rehabilitation service providers. “Standards for Reporting Qualitative Research (SRQR)” [29] guideline and “Critical Appraisal Skills Programme (CASP) Qualitative Checklist” [30] were considered to enhance the reporting and methodological quality. The institutional review board of Shiraz University of Medical Sciences (No. 27,348) approved the study’s protocol.

### Sampling and recruiting approach

In order to select participants, both convenient and snowball sampling methods were used. The interviewer (a female with a Ph.D. in physiotherapy) prepared a list of immigrants and refugees in need of PRS through communication with the centers that provide PRS to immigrants in Tehran, Isfahan, Fars, Sistan and Baluchistan, and Khorasan Razavi. She also prepared a list of PRS providers, including physiotherapists, occupational therapists, prosthesists/orthotists, audiologists, and optometrists, who served needed immigrants in these centers. For maximum diversity of participants, the research team selected participants from different places of residence, age groups, gender, education level, and occupation. In addition, interviewees were asked to introduce other potential individuals that could provide valuable information. Up until the participants came up with no new findings, sampling and interviewing continued. In order to ensure this achievement, the last three interviews with repeated findings were considered indicators of it [31]. After receiving the initial consent of the individuals to participate in the study, an informed consent form containing the general information of the research team as well as the research objectives was sent to them via email or instant messaging applications. Also, in this form, the participants were guaranteed that their identity would remain anonymous throughout the study and that they were free to withdraw from the study at any stage.

### Data collection

The first author conducted individual, semi-structured interviews in both face-to-face and online settings. An effort was made to conduct the interviews in a calm environment without the presence of a third party. Before initiating each interview, the interviewer again explained the general information and project objectives to the interviewee, and after obtaining her or his consent, the meeting started. During the interview sessions, an interview guide, including open questions prepared with the participation of all team members, was used (Table 1). Meanwhile, the questions in this interview guide were revised based on the feedback received from the initial interviews, with the aim of greater clarity. In order to facilitate the analysis of the collected qualitative data, the interviewer took notes during the interview sessions in addition to recording the sessions. At the end of each interview, the recorded file was written and saved in Office Word software.

**Table 1:** Interview guide

Open-ended questions
1. Express your experience of utilizing physical rehabilitation services by immigrants and refugees in Iran.
2. In your opinion, what factors make immigrants and refugees need physical rehabilitation services?
3. What factors increase the access of immigrants and refugees to physical rehabilitation services in Iran?
4. According to your experiences, what are the most important obstacles to benefiting from physical rehabilitation services by immigrants and refugees in Iran?
5. What are your suggestions to improve the use of physical rehabilitation services by immigrants and refugees in Iran?

### Data analysis

**Fig. 1:**
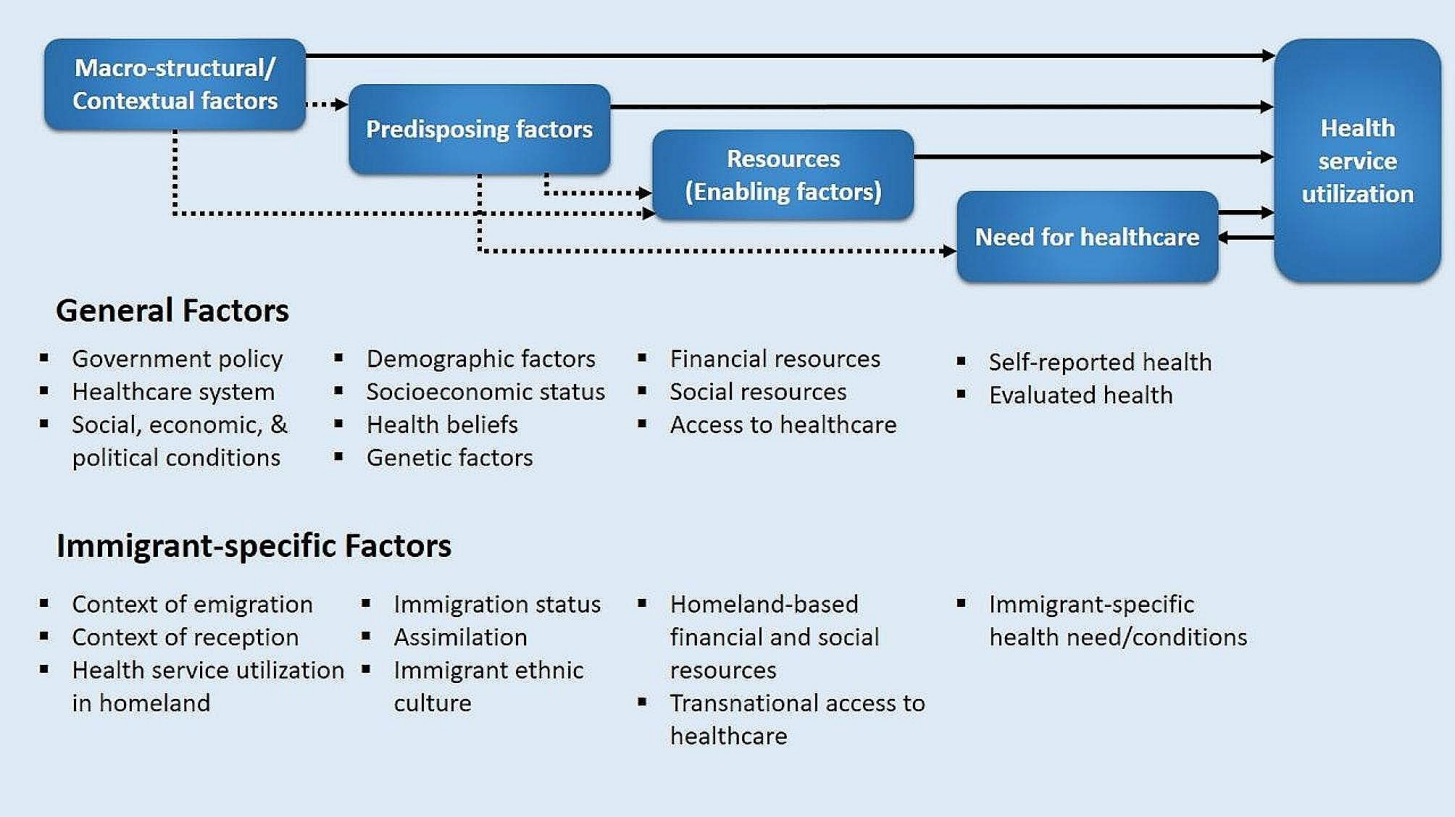
An analytical framework for immigrant health service utilization (Adopted from: Yang PQ, Hwang SH: Explaining immigrant health service utilization: a theoretical framework. Sage Open 2016, 6(2).)

The data analysis process was done simultaneously with the data collection and using the directed content analysis approach [32]. The components of the Immigrant Health Service Utilization (IHSU) Framework (Fig. 1), which was developed to indicate the special health utilization situation of immigrants [22], were considered pre-defined themes. This framework describes disparities in healthcare utilization among immigrants by need for healthcare, resources (enabling factors), predisposing factors, and macro-structural/contextual factors at both the immigrant-specific and general levels. Three members of the research team participated in the data analysis process. The transcribed texts were repeatedly reviewed, and the coding of the identified meaning units was done with the aim of developing the codebook. Then, the explored codes were synthesized and assigned inductively to the four main components of the adopted framework: (1) need for health care; (2) resources; (3) predisposing factors; and (4) macro-structural/contextual factors. As shown in Fig. 1, each of these components can be divided into general factors and immigrant-specific factors. Any differences of opinion among the authors were resolved at this stage through discussion and the participation of the expert author. Data analysis was conducted applying MAXQDA software (VERBI GmbH, Berlin, Germany).

### Rigor and trustworthiness strategies

To certify the rigor and trustworthiness of qualitative studies, a number of strategies are applied to enhance the authenticity, credibility, transferability, confirmability, and dependability of the findings [33]. In the current study, the research team considered several strategies to ensure rigor and trustworthiness: (a) inserting direct codes from almost all of the participants (authenticity); (b) immersing the authors in the study for the long term and member-checking by relevant experts (credibility); (c) selecting the participants with the highest diversity (transferability); (d) checking the final findings by participants (confirmability); and (e) involving authors with different scientific and executive backgrounds in the data analysis process (dependability).

### Ethical considerations

The ethical committee of the Shiraz University of Medical Sciences approved the study (IR.SUMS.REC.1401.674). Individuals were knowledgeable that their involvement in this study was voluntary and that they could give up the study at any time. An informed consent form was received from participants before each interview session.

## Results

In total, 28 individuals, including 19 service users and nine service providers, participated in this study. The characteristics of the participants are shown in Table 2. In the following, the views of the participants regarding the factors leading to the increase in the demand for PRS by immigrants and refugees with disabilities are presented first, and then the common barriers and enabling factors of utilizing PRS by this group are presented (Tables 3 and 4).

**Table 2:** Characteristics of participants

Participant	Age	Gender	Marital status	Job	Residence status	Residence location	Type of disability
S01	26	Male	Single	-	No information	Tehran	Amputation
S02	11	Male	Single	Student	Documented	Tehran	Cerebral palsy
S03	57	Male	Married	Peddler	Documented	Tehran	Amputation
S04	35	Female	Single	-	Documented	Khorasan Razavi	Foot deformity
S05	25	Male	Single	Daily worker	Undocumented	Sistan and Bluchestan	Amputation
S06	5	Male	Single	-	Undocumented	Sistan and Bluchestan	Cerebral palsy
S07	61	Female	Married	-	Documented	Khorasan Razavi	Diabetic foot
S08	33	Male	Married	-	Documented	Isfahan	Spinal cord injury
S09	22	Female	Single	-	Documented	Tehran	Amputation
S10	3.5	Female	Single	-	Documented	Sistan and Bluchestan	Cerebral palsy
S11	7	Male	Single	-	Documented	Tehran	Cerebral palsy
S12	43	Male	Married	Beggar	Undocumented	Isfahan	Amputation
S13	28	Male	Single	Welder	Documented	Sistan and Bluchestan	Foot deformity
S14	78	Male	Married	-	Documented	Khorasan Razavi	Cerebral vascular accident
S15	10	Male	Single	-	No information	Shiraz	Cerebral palsy
S16	8	Male	Single	Student	Undocumented	Tehran	Leg length discrepancy
S17	56	Male	Married	Working in a shop	Undocumented	Tehran	Amputation
S18	66	Female	Married	-	Documented	Khorasan Razavi	Cerebral vascular accident
S19	25	Female	Single	-	Documented	Tehran	Cerebral palsy
SP1	35	Male	Married	Physiotherapist	-	Sistan and Bluchestan	-
SP2	32	Male	Married	Occupational therapist	-	Khorasan Razavi	-
SP3	41	Male	Married	Prosthetist/Orthotist	-	Tehran	-
SP4	29	Female	Single	Physiotherapist	-	Tehran	-
SP5	32	Male	Single	Prosthetist/Orthotist	-	Shiraz	-
SP7	34	Male	Married	Prosthetist/Orthotist	-	Khorasan Razavi	-
SP8	38	Female	Single	Physiotherapist	-	Isfahan	-
SP9	28	Female	Single	Physiotherapist	-	Shiraz	-

**Table 3:** Common barriers of utilizing physical rehabilitation services by Afghan immigrants and refugees in Iran

Themes	Sub-themes	Barriers	Participants
Resources	Financial resources	Inadequacy of insurance coverage	S1, S2, S3, S4, S5, S6, S7, S11, S12, S14, S15, S17, S18
		High cost of services	SP4, S3, S4, S7, S10, S18, S19
		High cost of transportation and accommodation	SP1, SP2, SP3, S2, S5, S6, S8, S11, S15, S17, S19
	Social resources	Lack of social networks	SP2
	Informational resources	Lack of knowledge about Iran’s health system	S12, S15, S17
		Lack of awareness about immigration-related laws	S15
		Lack of awareness about available supports	SP3, S12, S15, S17
		Lack of awareness regarding the benefits of PRS	SP5, SP8
		Language barriers	SP4
	Access to healthcare	Lack of access to PRS in remote areas	SP1, SP4, S18
		Shortage of rehabilitation workforces, especially in remote regions	SP1
		Low quality of services	SP1
		Delayed referral or failure to follow up on treatment	S2, S15
		Lack of patience among providers	S8, S13, S14, S15
Predisposing factors (**General factors**)	Demographic factors	Reluctance of females to be examined by male practitioners	SP2
		High household size, which limits the incapacity to pay for the health needs of each member	S7
	Socioeconomic status	High rate of poverty among immigrants	SP1
		Low education level	SP3, SP6
		Living in remote and deprived areas	SP5, S19,
		Dispersion of immigrants	SP2
		Lack of cultural competence	SP7, SP9
	Health beliefs	Poor health literacy	SP2
		Lack of knowledge about the responsibilities of service providers	S5
		Willingness to receive services from physicians	S6, S7, S8
Predisposing factors (**Immigrant-specific factors**)	Immigration status	Inability to stay in accommodation centers for undocumented immigrants	SP1
		Fear of arrest and deportation	SP1, SP4, SP5, S5
		Inability to use public facilities	S3
	Assimilation	Cultural mismatch	SP2
	Immigrant ethnic culture	Lack of trust in modern treatments	S4, S6, S8
Macro-structural/contextual factors	Government Policy	Lack of a comprehensive policy for providing PRS	SP2, S4
		Existence of strict rules regarding immigrants’ residence	S14, S15, S16
		Lack of detailed information about existing health-related laws	S2, S4
	Healthcare system	Low priority of PRS in the Iranian health system	SP2, SP5
		Major provision of PRS by the private sector	SP6
		High out-of-pocket for PRS	SP2
		Inadequacy of insurance coverage	SP2
		Lack of interdisciplinary teamwork	SP2
		Inadequate physical rehabilitation infrastructure in smaller towns	S19
		Long waiting lists in public physical rehabilitation centers	S6
		High workload of PRS providers	S7
	Social, economic, and political conditions	High inflation rate, which limits the ability to pay for PRS	SP5, S1, S3, S4, S6, S8, S10, S14, S15, S16, S19
		Lack of job opportunities, which limits access to insurance	S5
		Lack of job security and access to permanent insurance for immigrants	SP4
		Low income and low capacity to pay	S13, S9
		Low social support	S2, S3, S15
		Unsuitability of urban environments	S3

Abbreviations: PRS; physical rehabilitation services

**Table 4:** Enabling factors for utilizing physical rehabilitation services by Afghan immigrants and refugees in Iran

Themes	Sub-themes	Enabling factors	Participants
Resources	Financial resources	Strengthening insurance coverage	S16, S19
		Increasing public funding for PRS	SP6
		Using charities’ and NGOs’ financial resources	SP2, S1, S15, S16, S18
		Using the capacity of employers to improve the financial capacity of immigrants	S8
		Creating an insurance fund for immigrants	S11
		Targeting the allocation of financial resources	SP3
	Social resources	Social support by Iranians	S1, S7
		Establishing social networks among immigrants	S11, SP2
	Informational resources	Informing immigrants about available services	SP2, S15, S17
		Educational campaigns	SP7
		Using Afghan volunteers as interpreters	SP2
	Access to healthcare	Using mobile physical rehabilitation teams	SP1
		Improving the accessibility of physical rehabilitation centers	S1
		Respectful behavior of providers with immigrant patients	S1, S2, S3, S4, S18, S19
		Sufficient time allocation by providers to need assessment	S3
Predisposing factors (**General factors**)	Socioeconomic status	Having an educated family	SP3
	Health beliefs	Trust in physical rehabilitation professionals	SP2
Predisposing factors (**Immigrant-specific factors**)	Immigration status	Not considering immigration status when providing services	SP5
		Facilitating the registration process for immigrants	S12
	Assimilation	Facilitating cultural integration	S11, SP2
	Immigrant ethnic culture	Trust in modern interventions	SP7
Macro-structural/contextual factors	Government Policy	Comprehensive needs assessments	SP1, SP3
		Informing immigrants about existing laws on their access to health services, including PRS	SP3
		Facilitating job-related skills training for immigrants	SP3
		Separating health services from politics	SP4
		Facilitating the receipt of international financial aid	SP4
		Clarifying the role and duties of each stakeholder in PRS delivery to immigrants	S2, S8
		Improving inter-sectoral collaborations for providing PRS to immigrants	SP1, SP6
	Healthcare system	Considering a basic benefit package for immigrants	SP7
		Investment in preventive services	S1, SP3
		Facilitating the referral of immigrants to higher health service levels	SP7
		Training the practitioners regarding the health needs of immigrants	S11, SP1
		Community-based education to improve health literacy	SP3, S3
		Increasing immigrants’ awareness of available services and eligibility criteria	SP3, S1, S8
		Investment in modern technology for delivering PRS	SP3
		Effective evaluation and supervision of PRS providers	SP3
		Providing health services regardless of race, language, and religion	SP4
		Strengthening the participation of charities and non-governmental organizations in providing PRS	S2, S3, S4, S12, S13
		Creating an integrated immigrant information system	S2
	Social, economic, and political conditions	Facilitating immigrants’ employment	SP3
		Enhancing advocacy from social and political stakeholders to ensure income and jobs for immigrants	SP4
		Increasing the public’s awareness regarding the immigrants’ health status	SP5

Abbreviations PRS; physical rehabilitation services

### Need for physical rehabilitation services

The disastrous social and economic situation in Afghanistan has led to a significant population of Afghans without access to timely and quality health services. Therefore, many of them are affected by conditions and injuries that lead to permanent physical disabilities.

*“The inefficiency of the healthcare system in Afghanistan has led to the persistence of certain diseases like cerebral palsy, resulting in long-term physical disability.” [SP6]*.

A significant share of the participants stated that the decade-long protracted conflict in Afghanistan has led to a high prevalence of physical disabilities.

*“Due to bombings, mine explosions, killings, and other recent civil wars in Afghanistan, the number of cases with physical disability has considerably increased.” [S01]*.

In addition, due to the high rate of consanguineous marriages, malnutrition, and unsafe childbirth in Afghanistan, cases of disability among children are significant.

*“In children, due to consanguineous marriages, improper nutrition during the mother’s pregnancy, unsafe delivery, and other such cases, it brings disorders to babies and harms them.” [SP3]*.

Another possible factor in increasing the need for PRS among Afghan immigrants is accidents and injuries on the way to Iran.

*“Many immigrants with disabilities are injured along the way due to hardships and accidents, many of whom require PRS.” [SP5]*.

Most Afghan immigrants and refugees in Iran are engaged in construction and heavy work, which has led to a high prevalence of musculoskeletal disorders among them.

*“Work injuries (including electrocution, burns, and amputation with a device during work) are among the factors that create disability among immigrants.” [SP3]*.

### Barriers in utilizing physical rehabilitation services

#### Resources

In almost all the interviews, the participants mentioned the challenges related to financial resources as one of the most important factors in the lack of sufficient benefit from PRS in Iran. In this regard, inadequacy of insurance coverage, high costs of services, and high costs of transportation and accommodation were the main financial barriers.

*“Due to the lack of insurance, the need that our child has had since childhood, and the increase in prices, it is difficult to receive services, and sometimes there is a gap.” [S02]*.

Immigrants live scattered geographically and are therefore lacking a social network, which would allow those with disabilities to exchange their experiences.

*“Many immigrants live in remote and marginalized areas. Therefore, many PwD do not know each other and cannot benefit from each other’s experiences.” [SP2]*.

Another common challenge to benefiting from PRS among immigrants with disabilities in Iran was the lack of access to accurate and user-friendly information about treatment and rehabilitation processes. The participants believed that the lack of knowledge about the structure of Iran’s health system, immigrant legislation, and support, especially for immigrants with disabilities, had affected access to services.

*“We don’t have insurance. We don’t know where to go to get services!” [S12]*.

*“Because we don’t know much about the laws related to immigrants in Iran, it takes a long time to be able to access the services.” [S15]*.

In addition, since many immigrants with disabilities do not have sufficient knowledge of the effects of physical rehabilitation interventions, they are not very willing to receive and adhere to these services.

*“Many clients do not believe in PRS. They prefer to receive medical treatment rather than rehabilitative interventions.” [SP5]*.

Although the language of Afghan immigrants is the same as that of Iranians, some of them speak local dialects such as Pashto, which makes communication between them and the medical staff difficult. One of the PRS providers mentioned this issue:

*“A limited number, especially those who are Pashto, are facing language problems.” [SP4]*.

During the interview sessions, the participants always mentioned the unavailability of PRS in remote areas and the lack of experienced human resources in these areas. Also, participants mentioned the low quality of PRS in some remote areas.

*“PRS in Iran are mainly located in the centers of provinces and densely populated cities. Therefore, it is very difficult to access these services in remote areas. In addition, the quality of service is not very acceptable in deprived areas!” [SP1]*.

Such a situation has caused patients to be referred late, and the follow-up of their treatment and rehabilitation process is not very favorable. One of the participants stated:

*“The lack of PRS in our city has made us go to get these services on time. Even when we receive these services in other cities, it is difficult to follow our treatment process.” [S15]*.

### Predisposing factors

The participants believed that gender as a predisposing factor affects the utilization of immigrants with disabilities in PRS.

*“Many Afghan women are reluctant to be evaluated and rehabilitated by a male therapist.” [SP2]*.

Moreover, the financial ability and capacity of Afghan families in Iran to pay for PRS have significantly declined as a result of their high household sizes.

*“We Afghans often have large families. This issue has caused us to not have enough financial ability to receive services due to our limited income.” [S7]*.

Regarding socioeconomic status, a major proportion of individuals talked about the high rate of poverty and low education level among immigrants, especially disabled immigrants.

*“Many of our clients are at risk of poverty. Well, it is clear that they cannot apply for PRS.”[SP1]*.

*“The level of education among most immigrants is very low. This factor has caused them to be reluctant to receive these services.”[SP3]*.

The disruption of the immigrants’ rehabilitation process was greatly attributed to their low health literacy and lack of awareness regarding the role and responsibilities of rehabilitation professions.

*“Many clients expect us to treat them like physicians. They expect to receive medicine, injections, and such interventions.”[SP2]*.

Throughout the study, participants pointed out that immigrant-specific risk factors that can affect PRS use by disabled immigrants include: (1) immigration status (not being able to stay in accommodation centers, fearing arrest and deportation, and not being able to use public facilities); (2) assimilation (cultural mismatch); and (3) immigrant ethnic culture (not trusting modern treatments).

*“Many immigrants who live in the country illegally are not allowed to attend the residence centers. This issue has caused many of them not to come to a city like Mashhad to receive services.” [SP1]*.

### Macro-structural/contextual factors

Macro-structural/contextual factors include general factors (government policy, healthcare system, social, economic, and political conditions) and immigrant specific factors (context of emigration, assimilation, and health service utilization in the homeland).

Regarding government policy, a number of participants believed that there was no comprehensive policy for providing PRS in Iran.

*“In Iran, various institutions provide PRS. It is not easy to benefit from these services, even for Iranians. In fact, there is no documented and detailed plan for the effective provision of these services in Iran.”[SP2]*.

In addition, some immigrants stated that although there are various laws related to the importance of their utilization of health services in Iran, these laws are strict and not very facilitating.

*“Although according to the law, we can receive health services, when receiving services, we have to present our residence documents, which makes many Afghans refuse to receive services.” [S14]*.

The participants pointed out that because there is not enough information about the available PRS and how to benefit from them, many immigrants in need cannot benefit from these services.

*“We don’t know much about the services available and how we can benefit from them.” [S4]*.

There are also various challenges in Iran’s health system that make it difficult for immigrants to benefit from PRS.

*“Rehabilitation problems not only for immigrants but also for Iranians have not been among the priorities of health policymakers.” [SP2]*.

Additionally, the private sector in Iran provides a sizable portion of PRS. This issue, along with the small insurance coverage of PRS, has caused the out-of-pocket payment to be very high when receiving services.

*“Many PRS in Iran are provided by the private sector, and since the insurance coverage of these services is not significant in Iran, the recipient is forced to pay almost all costs out of pocket. This issue is difficult for immigrants who do not have much income.” [SP2]*.

On the other hand, receiving PRS, especially in government and charity centers, is associated with long waiting lists.

*“Sometimes the waiting lists are so long that we give up getting services.” [S6]*.

The high workload of physical rehabilitation professions was another challenge that was mentioned during the interview sessions. One of the participants said:

*“They [service providers] are very busy, and we cannot ask them questions, and we have to wait in line.” [S7]*.

Many participants believed that Iran’s economic situation, especially high inflation, has greatly affected immigrants’ use of health services, especially PRS.

*“Everything is becoming more expensive in Iran. This has caused us to no longer have the capacity to receive these services.” [S1]*.

In addition, there is no significant social support for immigrants and refugees to benefit from health services, including PRS, in Iran.

*“There are not many support conditions here for people like me.” [S3]*.

In addition to all these cases, the infrastructure in urban environments affects the mobility of potential rehabilitation users.

*“Because I am in a wheelchair, it is very difficult to get around. It is difficult to cross the street, even if it is not far.” [S3]*.

### Enabling factors in utilizing physical rehabilitation services

#### Resources

The participants believed that strengthening financial resources could significantly improve their use of PRS. In this regard, several enabling factors were proposed during the interviews, including strengthening insurance coverage, increasing public funding, using charities’ and NGOs’ financial resources, using the capacity of employers, and creating an insurance fund for immigrants.

*“Even immigrants who have a residence permit and insurance have to pay most of the costs themselves when receiving services. It is necessary to increase the insurance coverage to receive more services.” [S16]*.

*“The financial capacity of non-governmental organizations and charities can be used to finance the PRS needed by immigrants.” [SP2]*.

One of the PRS providers stated that even if the necessary financial resources are available, it is necessary to allocate these resources in a targeted manner in order to avoid their waste.

*“Usually, in many countries, even when domestic and foreign financial resources are provided to provide health services for groups such as immigrants, they are not used effectively. Therefore, it is necessary to consider a targeted process in the allocation of designated resources.” [SP3]*.

The existence of social support from both Iranians and immigrant networks can significantly facilitate the benefits of PRS for immigrants with disabilities.

*“The neighbors (Iranians) guided us and said that centers like the Red Cross can help you.” [S7]*.

Regarding informational resources, participants believed that providing information about available services and holding educational campaigns about PRS could increase the use of these services.

*“There is a need to provide accurate information about the available services.” [SP2]*.

Afghan interpreters can also be used for groups that are not fluent in Persian in order to facilitate communication with the physical rehabilitation staff.

*“For immigrants who cannot speak Iranian Farsi, Afghans who have a longer residence history in Iran can be used to facilitate communication with service providers.” [SP2]*.

The participants stated that various factors can facilitate the access of immigrants with disabilities to PRS, including the use of mobile rehabilitation teams and improving access to existing rehabilitation centers.

*“Because many of these groups [disabled immigrants] live in remote areas and slums, mobile rehabilitation teams can be created to provide them with essential services.” [SP1]*.

In addition, it is necessary that the behavior of providers be such that disabled immigrants have more motivation to refer. The participants believed that the respectful behavior of the providers, as well as allocating enough time to examine and rehabilitate them, was very effective in helping them benefit from these services.

*“The providers treat us very well and spend enough time with us; this is very pleasant for us.” [S3]*.

### Predisposing factors

In relation to socio-economic status, one of the providers stated that having an educated family can partially facilitate the use of PRS, especially for children.

*“Immigrants with more educated families are more likely to apply for services.” [SP3]*.

Also, users’ trust to PRS providers enhances the referral and also their adherence to the treatment and rehabilitation process.

*“People who trust these services and providers tend to be more adherent to prescribed interventions.” [SP2]*.

According to the findings, not considering the residence status of immigrants can increase their benefits from services. One of the service providers stated:

*“I don’t think the residence status of immigrants should be asked when providing services. Because anyway, a significant share of them are undocumented.” [SP5]*.

Furthermore, it is necessary to facilitate and speed up the registration process for newly arrived immigrants.

*“Our [immigrants] registration process in Iran takes a lot of time. I think it should facilitate our registration process.” [S12]*.

Regarding assimilation, the participants stated that it is necessary to provide the necessary platform for cultural integration for immigrants.

*“Although Afghans and Iranians are two neighboring countries, there are still cultural differences that need to be addressed for the cultural integration of immigrants.” [SP2]*.

Many Afghan immigrants do not have much faith or trust in modern medical interventions. Therefore, after becoming disabled, they turn to local interventions, which worsen their health condition. In response, it is necessary to increase their level of awareness and trust in these services by using educational packages.

*“Many immigrants do not pay attention to the interventions provided by us. Most of them try to treat and rehabilitate using their religious and local beliefs.” [SP7]*.

### Macro-structural/contextual factors

During the interview sessions, participants mentioned several enabling factors related to government policy. They believed that providing PRS to disabled immigrants requires knowing the level of need for these services among immigrants.

*“A comprehensive needs assessment should be done at the country level so that policy- and decision-makers are fully aware of the demand for these services among refugees and immigrants.” [SP1]*.

In addition, information about existing laws and regulations related to the benefits of immigrants and refugees to health services, including PRS, should be provided accurately and continuously.

*“A mechanism should be provided so that this group [immigrants] has enough knowledge about the existing laws to access services.” [SP3]*.

The interviewees stated that providing job skills training courses for immigrants can improve their use of health services, including PRS, while strengthening their economic status. Also, while separating the process of providing health services from politics, efforts should be made to attract international financial resources in order to provide health services to these people.

*“Annually, significant financial resources are allocated by international organizations to improve the health of immigrants and asylum seekers worldwide. I think we should also try to attract these financial resources.” [SP4]*.

Clarifying the role of each stakeholder in providing PRS to immigrants as well as promoting interdepartmental colleagues were among the other suggestions presented in this study to increase the benefits of PRS for immigrants.

Regarding the healthcare system, participants, especially PRS providers, believed that a specific service package should be determined for immigrants and that major investment should be focused on services with preventive effects. In addition, the referral of disabled immigrants to other levels of the health system should be facilitated.

*“It should be possible to create a referral system so that needy immigrants can easily access other levels of the health system to follow up on their rehabilitation process.” [SP7]*.

Other suggestions provided by the participants in this category include the following: training the practitioners regarding the health needs of immigrants; community-based education to improve health literacy; increasing immigrants’ awareness of available services and eligibility criteria; and investment in modern technology in delivering PRS. Also, one of the providers believed that in order to provide quality services, it was necessary to have a strict evaluation and monitoring system for PRS.

*“In order to provide adequate quality services, they must be continuously evaluated and monitored.” [SP3]*.

During the interviews, it was stated that the participation of charities and non-governmental organizations in providing PRS can be significantly effective.

*“There are many domestic and international charities and NGOs that are interested in providing such services to vulnerable groups, such as immigrants.” [S4]*.

Efforts to stabilize the employment of immigrants, including the certification of health service providers, were intended to strengthen the economic status of immigrants. They believed that such conditions would increase their use of PRS.

*“Until immigrants’ employment and income status is resolved, they will be reluctant to use PRS, which is costly.” [SP3]*.

Also, it was stated that increasing the awareness and sensitivity of the general society towards the health status of immigrants and refugees can facilitate the use of services by immigrants.

*“In my opinion, the general society and especially policymakers should be sensitive to the health status of immigrants and its effect on other members of society so that these services can be provided for them.” [SP5]*.

## Discussion

The findings shed light on several key factors affecting access to PRS, including immigrants’ chronic problems, a lack of financial and social resources, and inadequate insurance coverage. Also, risk factors like gender, cultural inaccessibility, and a large household size, which makes it hard to pay, as well as immigrant-specific factors like immigration status, assimilation, and immigrant ethnic culture, played a big role in disabled immigrants’ use of PRS. The need for healthcare was identified as one of several factors inhibiting the use of PRS. Many disabled immigrants faced preexisting and emerging health conditions, which made them most in need of rehabilitation services. Chronic, long-term conditions, as well as new and emerging diseases, are common among the refugees. Half of the American adult refugee sample had at least one chronic NCD (51.1%) [34].

### Barriers in utilizing PRS among immigrants and refugees with disabilities

Immigrants are among the most in need of rehabilitation care, yet they are often the least able to access and use culturally adapted services due to social, economic, and political barriers [35]. Weak communication with healthcare providers, difficulties with recording refugees’ health data, tradition and culturally related aspects of healthcare-seeking behavior, and some language barriers are reported as the main barriers to accessing health services in Iran [36]. Furthermore, the lack of financial and social resources proved to be a major barrier, as disabled immigrants encountered challenges related to high costs of services, transportation, and accommodation. Insurance coverage was inadequate, and for some of them it doesn’t exist, leading to substantial out-of-pocket expenses, particularly in the private sector, where a significant portion of PRS in Iran is provided. Other studies also suggested that cost is the principal barrier to care-seeking for the refugees, and an inequitable healthcare policy in terms of insurance coverage was cited as a major barrier to accessing care [37]. Poor health literacy and the lack of awareness of one’s right to healthcare; language and cultural differences; protection issues resulting from a lack of legal status; and an inability to afford healthcare due to inadequate livelihoods are among the barriers to accessing health services by immigrants in Malaysia [38].

#### Shortage of infrastructures and geographical barriers

Additionally, the unavailability of PRS in remote areas and a shortage of experienced human resources in these regions further limited access to PRS for immigrants with disabilities, while most of them reside in marginalized and deprived regions.

#### Cultural barriers

Predisposing factors, such as gender, cultural inaccessibility, and high poverty rates among immigrants, were also identified as significant barriers. Cultural mismatches and a lack of trust in modern treatments among immigrant ethnic cultures were also noted as influential factors impeding PRS utilization. Settlement in suburban areas with limited public transportation and a lack of linguistically, culturally, and gender-appropriate services negatively affected access to and use of healthcare services for refugees, as reported in several studies [39–41]. The lack of cultural adaptation, such as insufficient information, as well as the location of the rehabilitation center and language barriers, affected the accessibility of the services to immigrant families in Norway [42]. Barriers to establishing social networks and utilizing available disability services were reported among fathers with disabled children [43].

#### Political barriers

The specific immigration status of disabled immigrants contributed to their inability to access public facilities and their fear of arrest and deportation, which discouraged them from seeking PRS. Physical and other impairments will often make it more difficult for individuals to access safety and relief opportunities. However, PwD have been ignored for far too long [44].

#### Macro-structural level barriers

The lack of comprehensive policies for providing PRS, either for Iranians or immigrants in Iran, posed a substantial barrier. Health policymakers did not prioritize PRS, which resulted in a lack of knowledge about the services that were available and how to access them. The lack of knowledge about the structure of Iran’s health system, the laws related to immigrants, and the existing support for immigrants, especially immigrants with disabilities, has caused the level of service utilization by immigrants to be low. Complications in navigating the healthcare system are identified as a barrier in other countries [45] and health system barriers include external resource constraints, costs to the individual, discrimination, and high bureaucratic requirements [46].

Government and charity centers faced long waiting lists for PRS, hindering timely access. Moreover, the high workload of physical rehabilitation professionals further strained the availability of services. Additionally, high inflation and unemployment rates pose significant challenges for immigrants and refugees with disabilities seeking PRS in Iran. These barriers are exacerbated in the cases of immigrants and refugees who are undocumented, which leads to their lack of legal access to healthcare services. A study that specifically targeted undocumented migrants revealed that 62% of them had unmet health needs, while 53% had major difficulties accessing health services. Key obstacles included cost-related issues and extended waiting lists [47].

### Enabling factors in utilizing PRS among immigrants and refugees with disabilities

Despite the barriers, several enabling factors were reported by participants to improve PRS utilization.

#### Financial support policies

Strengthening financial resources, insurance coverage, and public funding were proposed to reduce the financial burden on immigrants and refugees with disabilities seeking PRS. Addressing income inequality, improving access to social and developmental services, and improving the cultural sensitivity of services have been recommended in other studies [24]. Leveraging resources from charities, NGOs, and employers was also suggested to enhance accessibility.

#### Macro-structural level interventions

Improving information dissemination about available services and conducting educational campaigns were identified as potential enabling factors. Facilitating the referral process to other levels of the health system, training practitioners to understand immigrants and refugees health needs, promoting community-based education, and investing in modern technology were also recommended strategies. Tofani et al. have mentioned that removing barriers to rehabilitation and assistive technology for refugees with disabilities should focus on health literacy and the empowerment of migrants, data collection on health, disability, and assistive technology, and the organization of community-based rehabilitation programs [11]. A US-based study recommends an expanded pool of medical interpreters, peer navigators, innovative health information technologies, and greater collaboration and information sharing between service systems to address barriers affecting disabled and chronically ill refugees [48].

Providing job skills training courses and improving economic status were suggested to empower immigrants and refugees with disabilities to access PRS. Separating the provision of health services from politics could attract international financial resources to cater to the healthcare needs of immigrants and refugees. Collaboration among departments and investment in services with preventive effects were deemed beneficial. Additionally, the participation of charities and NGOs in providing PRS and efforts toward the stable employment of immigrants and refugees were considered valuable steps to enhance utilization. Host countries should assess the mental health of newcomers alongside physical evaluations, grant humanitarian migrants’ access to regular health care, and ensure they are able to use it [49].

#### Improving geographical access

Using mobile rehabilitation teams and improving access to existing centers could address the problem of unavailability in remote areas. Other study has shown that utilizing mobile rehabilitation teams and telerehabilitation could address the problem of unavailability in remote areas for immigrants and refugees [50].

### Cultural interventions

Moreover, improving the behavior of service providers and allocating sufficient time to each patient were considered crucial to promoting utilization. Addressing predisposing factors, such as facilitating the registration process for newly arrived immigrants and refugees and promoting cultural integration, was proposed to improve PRS utilization. Building trust in modern PRS through educational packages and increasing their literacy level were also highlighted as important enabling factors. Healthcare providers should help clients become more aware of the resources available to them in the hospital and in the community. More time should be allotted when working with immigrant and refugee families to build rapport [35].

### Limitations

This qualitative study is subject to certain limitations. Firstly, the study samples were drawn exclusively from the five provinces in Iran with the highest immigrant populations, which may limit the generalizability of the findings to a broader national context. Secondly, the interviews were conducted with providers and individuals who had visited rehabilitation centers of NGOs and national societies. However, it is important to acknowledge that a significant proportion of immigrants and refugees in need of PRS may not access these centers due to various factors, which were not explored within the scope of this study. This limited our ability to capture the perspectives of those who face barriers preventing them from seeking PRS. Consequently, the study’s findings may not fully represent the experiences and challenges of this underserved population. In response, future studies should investigate the barriers to accessing these services among all immigrants and refugees, not just the group that visits rehabilitation centers. In addition, quantitative studies are needed to monitor the real demands for receiving different types of PRS among immigrants and refugees in Iran.

## Conclusion

Our study shed light on the barriers to utilizing needed PRS among Afghan immigrants and refugees with disabilities and showed that there are multiple cultural, financial, social, and political barriers that make this group struggle to utilize PRS. Recognizing the multifaceted nature of these challenges and opportunities is not only crucial but imperative. By proactively addressing these factors, stakeholders can chart a path towards devising meticulously targeted interventions and formulating policies that foster equitable access and heightened utilization. The potential impact of such measures extends far beyond numerical improvements; it resonates in the tangible enhancement of health outcomes for this particularly vulnerable and underserved population. Nevertheless, acknowledging the complexity of their experiences and the dynamic nature of this field underscores the ongoing importance of research and collaboration to continually refine and adapt these interventions, ensuring a responsive and inclusive healthcare landscape for all. The international and national authorities should push access to PRS services for the refugees on the agenda to make sure, with the high rate of disability among these vulnerable people, access to high-quality, affordable PRS services is well defined and introduced to this community.

## Data Availability

The datasets used and/or analyzed during the current study are available from the corresponding author on reasonable request.

## References

[1] Koczan Z, Peri G, Pinat M, Rozhkov D. The impact of international migration on inclusive growth: A review. IMF Working Paper No. 2021/088, Available at SSRN: https://ssrn.com/abstract=4026261 2021.

[2] Gushulak BD, Weekers J, MacPherson DW (2009). Migrants and emerging public health issues in a globalized world: threats, risks and challenges, an evidence-based framework. Emerg Health Threats J.

[3] Elsby MW, Smith J, Wadsworth J. Population growth, immigration and labour market dynamics. 2021. 10.1215/00703370-11579897 39324837

[4] Buchan J, Campbell J, Dhillon I, Charlesworth A. Labour market change and the international mobility of health workers. Health Foundation working paper. 2019, 5.

[5] Brennan N, Langdon N, Bryce M, Burns L, Humphries N, Knapton A, Gale T (2023). Drivers and barriers of international migration of doctors to and from the United Kingdom: a scoping review. Hum Resour Health.

[6] Shahabi S, Skempes D, Pardhan S, Jalali M, Mojgani P, Lankarani KB (2021). Nine years of war and internal conflicts in Syria: a call for physical rehabilitation services. Disabil Soc.

[7] Boggs D, Atijosan-Ayodele O, Yonso H, Scherer N, O’Fallon T, Deniz G, Volkan S, Örücü A, Pivato I, Beck AH (2021). Musculoskeletal impairment among Syrian refugees living in Sultanbeyli, Turkey: prevalence, cause, diagnosis and need for related services and assistive products. Confl Health.

[8] World Health Organization. 2023. Geneva: World Health Organization. Accessed here: https://www.who.int/news-room/fact-sheets/detail/rehabilitation.

[9] United Nations High Commissioner for Refugees. Global forced displacement tops 70 million. 2019. Accessed here: https://www.unhcr.org/news/stories/global-forced-displacement-tops-70-million.

[10] UN. Thematic study on the rights of persons with disabilities under article 11 of the Convention on the Rights of Persons with Disabilities, on situations of risk and humanitarian emergencies. In.: A/HRC/31/30; 2015.

[11] Tofani M, Iorio S, Berardi A, Galeoto G, Conte A, Fabbrini G, Valente D, Marceca M (2023). Disability, Rehabilitation, and Assistive Technologies for Refugees and Asylum Seekers in Italy: policies and challenges. Societies.

[12] Burnett A, Peel M (2001). Health needs of asylum seekers and refugees. BMJ.

[13] Al-Oraibi A, Hassan O, Chattopadhyay K, Nellums L (2022). The prevalence of non-communicable diseases among Syrian refugees in Syria’s neighbouring host countries: a systematic review and meta-analysis. Public Health.

[14] Divkolaye NSH, Burkle FM Jr. The enduring health challenges of Afghan immigrants and refugees in Iran: a systematic review. PLoS Curr 2011, 9. 10.1371/currents.dis.449b4c549951e359363a90a7f4cf8fc4 PMC5554007 28856065

[15] Khan F, Amatya B (2017). Refugee health and rehabilitation: challenges and response. J Rehabil Med.

[16] Oner O, Kahilogullari AK, Acarlar B, Malaj A, Alatas E (2020). Psychosocial and cultural needs of children with intellectual disability and their families among the Syrian refugee population in Turkey. J Intellect Disabil Res.

[17] King J, Edwards N, Correa-Velez I, Hair S, Fordyce M (2016). Disadvantage and disability: experiences of people from refugee backgrounds with disability living in Australia. Disabil Global South.

[18] Schröder CC, Breckenkamp J, du Prel JB (2022). Medical rehabilitation of older employees with migrant background in Germany: does the utilization meet the needs?. PLoS ONE.

[19] Kim KM, Hwang SK (2019). Being a ‘good’mother: immigrant mothers of disabled children. Int Social Work.

[20] Khanlou N, Haque N, Sheehan S, Jones G (2015). It is an issue of not knowing where to go: Service Providers’ perspectives on challenges in accessing social support and services by Immigrant Mothers of Children with disabilities. J Immigr Minor Health.

[21] Zimba O, Gasparyan AY. Refugee Health: A Global and Multidisciplinary Challenge. J Korean Med Sci 2023, 38(6). 10.3346/jkms.2023.38.e60 PMC9925328 36786089

[22] Yang PQ, Hwang SH (2016). Explaining immigrant health service utilization: a theoretical framework. Sage Open.

[23] Dew A, Lenette C, Wells R, Higgins M, McMahon T, Coello M, Momartin S, Raman S, Bibby H, Smith L (2023). In the beginning it was difficult but things got easier’: Service use experiences of family members of people with disability from Iraqi and Syrian refugee backgrounds. J Policy Pract Intellect Disabil.

[24] Khanlou N, Mustafa N, Vazquez LM, Haque N, Yoshida K (2015). Stressors and barriers to services for immigrant fathers raising children with Developmental Disabilities. Int J Mental Health Addict.

[25] Dyck M, Breckenkamp J, Wicherski J, Schröder CC, du Prel J-B, Razum O (2020). Utilisation of medical rehabilitation services by persons of working age with a migrant background, in comparison to non-migrants: a scoping review. Public Health Rev.

[26] Haar RJ, Wang K, Venters H, Salonen S, Patel R, Nelson T, Mishori R, Parmar PK. Documentation of human rights abuses among rohingya refugees from Myanmar. Confl Health 2019, 13(1). 10.1186/s13031-019-0226-9 PMC6745767 31534473

[27] Polack S, Scherer N, Yonso H, Volkan S, Pivato I, Shaikhani A, Boggs D, Beck AH, Atijosan-Ayodele O, Deniz G (2021). Disability among Syrian refugees living in Sultanbeyli, Istanbul: results from a population-based survey. PLoS ONE.

[28] Norredam M, Nielsen SS, Krasnik A (2010). Migrants’ utilization of somatic healthcare services in Europe—a systematic review. Eur J Pub Health.

[29] O’Brien BC, Harris IB, Beckman TJ, Reed DA, Cook DA (2014). Standards for reporting qualitative research: a synthesis of recommendations. Acad Med.

[30] Long HA, French DP, Brooks JM (2020). Optimising the value of the critical appraisal skills programme (CASP) tool for quality appraisal in qualitative evidence synthesis. Res Methods Med Health Sci.

[31] Saunders B, Sim J, Kingstone T, Baker S, Waterfield J, Bartlam B, Burroughs H, Jinks C (2018). Saturation in qualitative research: exploring its conceptualization and operationalization. Qual Quant.

[32] Assarroudi A, Heshmati Nabavi F, Armat MR, Ebadi A, Vaismoradi M (2018). Directed qualitative content analysis: the description and elaboration of its underpinning methods and data analysis process. J Res Nurs.

[33] Kyngäs H, Kääriäinen M, Elo S. The trustworthiness of content analysis. The application of content analysis in nursing science research. edn.: Springer; 2020. pp. 41–8.

[34] Yun K, Hebrank K, Graber LK, Sullivan M-C, Chen I, Gupta J (2012). High prevalence of chronic non-communicable conditions among adult refugees: implications for practice and policy. J Community Health.

[35] Lindsay S, King G, Klassen AF, Esses V, Stachel M (2012). Working with immigrant families raising a child with a disability: challenges and recommendations for healthcare and community service providers. Disabil Rehabil.

[36] Rahimitabar P, Kraemer A, Bozorgmehr K, Ebrahimi F, Takian A. Health condition of Afghan refugees residing in Iran in comparison to Germany: a systematic review of empirical studies. Int J Equity Health 2023, 22(1). 10.1186/s12939-023-01832-7 PMC9862781 36681845

[37] Mohammadi S, Carlbom A, Taheripanah R, Essén B. Experiences of inequitable care among Afghan mothers surviving near-miss morbidity in Tehran, Iran: a qualitative interview study. Int J Equity Health 2017, 16(1). 10.1186/s12939-017-0617-8 PMC5500928 28687082

[38] Chuah FLH, Tan ST, Yeo J, Legido-Quigley H (2018). The health needs and access barriers among refugees and asylum-seekers in Malaysia: a qualitative study. Int J Equity Health.

[39] Guruge S, Sidani S, Illesinghe V, Younes R, Bukhari H, Altenberg J, Rashid M, Fredericks S (2018). Healthcare needs and health service utilization by Syrian refugee women in Toronto. Confl Health.

[40] BMC Pregnancy and Childbirth. 2020, 20(1). 10.1186/s12884-019-2700-1 PMC6945660 31906880

[41] Dadras O, Dadras F, Taghizade Z, Seyedalinaghi S, Ono-Kihara M, Kihara M, Nakayama T. Barriers and associated factors for adequate antenatal care among Afghan women in Iran; findings from a community-based survey. BMC Pregnancy Childbirth 2020, 20(1). 10.1186/s12884-020-03121-5 PMC7389441 32723332

[42] Arfa S, Solvang PK, Berg B, Jahnsen R (2022). Participation in a rehabilitation program based on adapted physical activities in Norway: a qualitative study of experiences of immigrant parents and their children with disabilities. Disabil Rehabil.

[43] Alsharaydeh E, Alqudah M, Lee R, Chan S (2023). Challenges, coping and resilience in caring for children with disability among immigrant parents: a mixed methods study. J Adv Nurs.

[44] Smith-Khan L, Crock M (2019). The highest attainable standard’: the right to health for refugees with disabilities. Societies.

[45] Asif Z, Kienzler H (2022). Structural barriers to refugee, asylum seeker and undocumented migrant healthcare access. Perceptions of doctors of the World caseworkers in the UK. SSM-Mental Health.

[46] Hacker K, Anies M, Folb BL, Zallman L. Barriers to health care for undocumented immigrants: a literature review. Risk Manage Healthc Policy 2015:175–83. 10.2147/RMHP.S70173 PMC4634824 26586971

[47] Schottland-Cox J, Hartman J (2019). Physical therapists needed: the Refugee Crisis in Greece and our ethical responsibility to Respond. Phys Ther.

[48] Mirza M, Luna R, Mathews B, Hasnain R, Hebert E, Niebauer A, Mishra UD (2014). Barriers to healthcare access among refugees with disabilities and chronic health conditions resettled in the US Midwest. J Immigr Minor Health.

[49] Matlin SA, Depoux A, Schütte S, Flahault A, Saso L (2018). Migrants’ and refugees’ health: towards an agenda of solutions. Public Health Rev.

[50] Castillo YA, Cartwright J. Telerehabilitation in Rural Areas: A Qualitative Investigation of Pre-service Rehabilitation Professionals’ Perspectives. *sgrjarc* (2):6–13.

